# Digital frontiers in international psychiatric recruitment: the lessons of the Northwest School of Psychiatry careers event November 2020

**DOI:** 10.1192/bjo.2021.406

**Published:** 2021-06-18

**Authors:** Nosheen Kazmi, Catarina Rodrigues dos Santos, Emily Lewis, Sahana Olety

**Affiliations:** 1 ST4 CAMHS, Pennine Care NHS Foundation Trust; 2 ST4 Forensic Psychiatrist, Greater Manchester Mental Health; 3 Specialist Doctor in Psychiatry, Cheshire and Wirral Partnership NHS Foundation Trust; 4 Foundation Tutor, Pennine Care NHS Foundation Trust, Consultant Child and Adolescent Psychiatrist

## Abstract

**Aims:**

A low level of psychiatric recruitment is a global issue[1]. The RCPsych & UK Mental Health Trusts jointly run School Events as part of a recruitment strategy. The North West has been running such events for the past years. After our first virtual event, we compare the quality, effectiveness and experience of a face-to-face (F2F) recruitment programme in 2018 with our first remote recruitment programme in 2020. In a world of fast paced technology, we reflect on what lies in the horizon for international psychiatric recruitment.

**Method:**

The recruitment programme was organised by 4 psychiatric trainees affiliated to each mental health trust. A two-day remote programme on the Zoom platform comprising of 45-minute slots was created. Through their own experiences of inspirational speakers, trainees contacted speakers representing different specialities, teaching styles and philosophical outlooks.

Pre and Post Programme questionnaires and certificates of attendance were shared with speakers and attendees. These were compared with Pre and Post Programme questionnaires from the F2F event in 2018.

**Result:**

When compared to the 2018 F2F programme, the 2020 virtual recruitment programme attracted a higher number and wider variety of applicants, in gender (62% female/38%male), nationality (UK 79%/Non-UK 22%), as well as wider distribution in age, UK deanery and training position. Despite the elimination of cost, the quality of teaching was rated higher than F2F due to the availability of high quality speakers (100% would recommend to a friend; 72% rated excellent). Whilst the programme was effective in changing minds, this did not exceed F2F recruitment rates. F2F recruitment feedback focussed on inclusion of sub-specialities, whereas virtual programme feedback focussed on ways to enhance interaction. Feedback focussed on technological applications such as interactive quizzes, breakout rooms, play-acting, and having the benefit of clinical vignettes or speakers’ personal stories to bring talks to life.

**Conclusion:**

The use of remote technology transcended geographical and demographic frontiers. A variety of high-quality speakers, directly appealing to an international cohort were sourced, at no monetary cost. In future, the budget will be used in interactive applications, and time-limited session recordings. As the participants hungered for personal connections, we recommend a blended programme, with links to taster sessions, retaining the advantages of both strategies.

